# Preservation of Higher-Order Cognition in Autistic Females: Evidence from Two Deeply-Phenotyped Cohorts in Hong Kong and the United States

**DOI:** 10.21203/rs.3.rs-10617508/v1

**Published:** 2026-08-25

**Authors:** Oscar WH Wong, Sophia Zhu, Jonathan WT Chow, Ran Barzilay, Tyler M. Moore, Kosha Ruparel, Angela MW Lam, Iga Adamska-Stolarczyk, Petrina YP Lau, Sandra SM Chan, Monica E. Calkins, Raquel E. Gur, Ruben C. Gur

**Affiliations:** The Chinese University of Hong Kong; The Chinese University of Hong Kong; The Chinese University of Hong Kong; Children’s Hospital of Philadelphia and Penn Medicine; University of Pennsylvania; University of Pennsylvania; The Chinese University of Hong Kong; The Chinese University of Hong Kong; The Chinese University of Hong Kong; The Chinese University of Hong Kong; University of Pennsylvania; University of Pennsylvania; University of Pennsylvania

**Keywords:** Autism spectrum disorder, sex differences, cognition, female, executive function, higher-order cognition, compensation, camouflage

## Abstract

**Background:**

Sex differences in the clinical presentation of autism spectrum disorder (ASD) are well documented. Although prior studies have linked neurocognitive differences to these phenotypic variations, it remains unclear whether such differences are specific to ASD or attributable to co-occurring psychopathology and intrinsic sex differences in the general population.

**Methods:**

This study evaluated multi-domain cognition with the Penn Computerized Neurocognitive Battery and clinical assessments in 1,101 Chinese children in Hong Kong (701 with ASD, 400 without ASD; aged 6–12 years), with concurrent analysis in an independent US cohort of 1,090 participants (218 with ASD, 872 without ASD; aged 8–21 years with diverse ethnicity). Group differences were analysed using MANCOVA followed by univariate ANCOVA, with rigorous adjustment for demographics and co-occurring psychopathology.

**Results:**

Across both cohorts, a consistent Group × Sex interaction was observed specifically in the cognitive domain Complex Reasoning and Executive Function (CREF), with boys with ASD showing the greatest impairment, while girls with ASD performed comparably to non-ASD girls. Exploratory analyses in the Hong Kong cohort, stratifying ASD into ASD-only and ASD+ADHD subgroups, revealed a stepwise significant decline in CREF among boys (non-ASD > ASD-only > ASD+ADHD). In girls, CREF was largely preserved in the ASD-only group relative to non-ASD controls, while the ASD+ADHD subgroup showed lower performance that did not reach statistical significance after correction for multiple comparisons.

**Conclusions:**

These findings indicate that relatively preserved higher-order cognition in autistic females may help explain subtler clinical presentations observed in some females with ASD. Given evidence linking higher-order cognition to compensatory processes, our results have implications for improving clinical detection of autistic females, addressing their specific needs, and understanding biological mechanisms underlying sex differences in ASD.

## Background

Autism spectrum disorder (ASD) has long been recognized as a male-predominant condition, with historical male-to-female ratios reported as high as 4:1. However, more recent estimates closer to 3:1 have raised the possibility of diagnostic bias against females [1]. Over the past decade, systematic investigation has revealed a distinct female autism phenotype, characterized by qualitative and quantitative differences in behavioural presentation. For example, restricted and repetitive behaviors (RRB) in autistic females may manifest as idiosyncratic language or perfectionism rather than overt motor stereotypies, while social difficulties may be masked by superficially intact conversational skills and nonverbal communication [2]. Diagnostic instruments commonly used in epidemiological studies (such as Autism Diagnostic Interview-Revised, ADI-R and Autism Diagnostic Observation, ADOS) were developed primarily based on male prototypes, potentially contributing to under-recognition of autistic females at the population level [3].

Beyond phenotypic presentation, sex differences are also evident in symptom trajectories and prognosis. Girls with autism often show greater symptom improvement in childhood compared to boys [4], which has prompted investigations into the biology that drives the clinical divergence. From a genetic perspective, females appear to have a higher threshold for developing ASD despite carrying comparable genetic liability as males – a phenomenon known as the female protective effect. However, the underlying mechanisms of this protective effect remain largely unknown [5]. Curiously, the male-to-female ratio shifts dramatically from approximately 6–16:1 in individuals without intellectual disability, to nearly 1–2:1 among those with co-occurring severe intellectual disability [6]. This pattern suggests that the female protective effect may be attenuated when cognitive impairment is profound, raising the possibility that cognitive functioning could be one of the mechanisms contributing to this protective effect.

Children with ASD were often shown to have poorer performance at group level than non-ASD children across multiple cognitive domains [7–9], despite the substantial individual variability in cognitive abilities within the autism spectrum [10]. Cognitive abilities are strong correlates to the clinical severity and prognosis of ASD [11, 12]. Among cognitive domains, executive function (EF) and higher-order cognitions appear particularly relevant, as they serve as compensatory mechanisms and facilitate camouflaging strategies that are more commonly observed in autistic females [13, 14]. Differences in these higher-order cognitive functions and EF may therefore contribute to the distinct clinical presentations and trajectories between autistic males and females.

The literature on cognitive sex differences in ASD has yielded mixed results. Some studies report poorer overall cognitive performance in females with ASD [15], while others find no significant sex differences [16]. Another study suggested a female advantage in specific domains such as verbal fluency [17]. These inconsistent findings could arise from several methodological variations and limitations. For instance, several studies examined cognitive domains in isolation [18, 19], making it difficult to determine whether observed effects were domain-specific given the high intercorrelations among cognitive abilities. Additionally, the confounding impact of co-occurring psychopathology has not been adequately addressed in several studies [16, 20], given the association of psychopathology with cognitive performance [12, 21]. Additionally, generalized slower reaction times are well-documented in ASD and may confound cognitive performance measures [22]. Furthermore, only a minority of studies included a non-ASD control group [23, 24], even though intrinsic sex differences in cognitive abilities are well-documented in the general population [25, 26]. Without such controls, it would be unclear whether observed differences merely reflect typical sex differences.

Here, we first examined sex differences in multi-domain cognitive performance using a validated computerized neurocognitive battery in Chinese autistic and non-autistic children in Hong Kong aged 6–12 years. We applied rigorous control for co-occurring psychopathology and other potential confounders. We then sought to replicate key findings in an independent US cohort with an older and broader age range (8–21 years) and ethnic diversity, assessed by the same neurocognitive battery and comparable clinical deep phenotyping.

## Method

### Study Population

#### Hong Kong Cohort (HATCH cohort)

Participants were from the Hub of Advanced Technology for Child Health (HATCH) Cohort [27]. Chinese children aged 6–12 years with ASD (*n* = 701) were recruited from a university-affiliated psychiatric center in Hong Kong. Diagnoses of ASD were made by child psychiatrists according to the Diagnostic and Statistical Manual of Mental Disorders, Fifth Edition (DSM-5) [28]. Although participants were recruited from a specialist psychiatric clinic, the clinic serves as the main referral center for ASD in the region and is closely linked to primary care screening pathways. It provides both diagnostic services and training interventions, thereby capturing a broad spectrum of ASD presentations, including children with and without significant co-occurring conditions. Non-autistic children of the same age range (*n* = 400) were recruited from a local psychiatric epidemiological study and screened negative for autism using the Hong Kong version of the 10-item Autism Spectrum Quotient (AQ-10-HK) [29], as well as for the presence of 31 DSM-5-defined psychiatric disorders with the Diagnostic Interview Schedule for Children Version 5 (DISC-5) [30]. For both groups, individuals with intellectual disability, neurological disorders, other major medical illnesses, and psychiatric conditions that are rare in prepubescent children (such as psychosis and depressive disorders) were excluded. The study was approved by the Joint Chinese University of Hong Kong-NTEC Clinical Research Ethics Committee (Ref.: 2021.550). Written informed consent was given by parents or guardians, while assent was obtained for all participating children.

##### United States Philadelphia Neurodevelopmental Cohort (US PNC)

Participants for the replication study were drawn from the Philadelphia Neurodevelopmental Cohort (PNC), a large community-based study of 9,498 youths aged 8–21 years recruited from pediatric clinics within the Children’s Hospital of Philadelphia healthcare network, all stable health and without significant physical conditions or developmental delay impairing ability to participate in study procedures [31]. As participants were recruited from pediatric rather than psychiatric clinics, the sample was not enriched for individuals seeking psychiatric care. Youths with ASD (*n* = 218) were identified through self- or parent-report and electronic health record verification. A matched comparison group (*n* = 872) was selected at a 4:1 ratio on age, sex, race, and socioeconomic status. While a recent analysis of the same PNC sample examined broader sex differences in psychopathology and social versus non-social cognition [32], the present study focuses specifically on domain-level cognition—particularly higher-order cognition—and tests generalizability of results from the Hong Kong HATCH Cohort. Participants and their guardians (for participants < 18 years) provided written informed consent or assent. All procedures were approved by the University of Pennsylvania and the Children’s Hospital of Philadelphia Institutional Review Boards.

##### Clinical Profiling

###### Hong Kong HATCH Cohort

Core ASD features were assessed using the Chinese versions of the Social Responsiveness Scale-2 (SRS-2) [33]. Given the high prevalence of co-occurring ADHD in this age range [34], ADHD was systematically evaluated in every child with ASD at the psychiatric centre by child psychiatrists according to the DSM-5 criteria. Dimensional psychopathologies were further evaluated using the Attention Problems Subscales from the Child Behavior Checklist (CBCL) [35], and the Anxiety Scale for Children-ASD (ASC-ASD) [36]. The short form of the Hong Kong Wechsler Intelligence Scale for Children, 4th Edition [WISC-IV (HK) Short form] [37] was used to measure the Full-scale Intelligence Quotient (FSIQ) of the participants with ASD.

###### US PNC

While the PNC did not have specific dimensional measures of ASD symptomatology, psychopathology was comprehensively assessed using the Grand Opportunity Assessment (GOASSESS) structured interview [31]. A correlated factor model was implemented on the 112 item-level symptoms from the GOASSESS to generate four dimensional psychopathology factors, namely anxious-misery, fear, psychosis, and externalizing behavior [21, 38]. Estimated IQ was obtained using the Wide Range Achievement Test (WRAT-4) Reading subscale.

##### Neurocognitive Assessment

Multidomain neurocognitive functioning was assessed using the Penn Computerized Neurocognitive Battery (CNB). In the PNC, the original English version of the CNB was used. In the HATCH cohort, a culturally adapted Hong Kong version (CNB-HK) was administered. Both versions consisted of 12 domain-specific tasks plus a motor praxis task that measures sensorimotor processing speed [39]. The CNB has an administration time of approximately one hour. Eleven of the domain-specific tasks were identical between the CNB and CNB-HK. The logical reasoning task was omitted from the CNB-HK due to challenges in accurate translation into Chinese, and a Go/No-Go task was adopted in the CNB-HK. For a complete list of tasks in both versions, see Supplementary Table S1. The domain-specific tasks were scored on accuracy, while the motor praxis task was scored on speed. All scores were z-transformed according to age-standardized norms. Accuracy z-scores from the domain-specific tasks were summarized into three factors — Episodic Memory, Social Cognition, and Complex Reasoning & Executive Function (CREF) — using a correlated factor model. The cultural adaptation and translation of the CNB-HK, administration procedures, derivation of factor scores, and psychometric properties of both the CNB and CNB-HK have been described in detail previously [40, 41]. The factor structures of the two versions were highly comparable, supporting the validity of cross-cohort analyses.

Neurocognitive assessments were conducted in private research offices by trained research staff. A standardized CNB administration manual was followed, with written instructions that were read aloud and trial sessions prior to each task to ensure participants’ understanding. The sequence of tasks was predetermined, and standardized ratings were used to evaluate the completeness and validity of the data collected. For each participant, test administrators also recorded any scenarios that would possibly affect data integrity. There were regular meetings to confirm scoring validity. For each task, data that are incomplete or with validity concerns were discarded. In the HATCH cohort, autistic children with co-occurring ADHD who were prescribed psychostimulants were asked to withhold medication on the day of CNB-HK administration whenever feasible. In cases where parents judged that withholding medication would render the child too hyperactive or inattentive to complete the assessment, stimulant use was permitted, and this information was recorded for subsequent statistical adjustment as a covariate.

#### Statistical Analysis

Statistical analyses were performed using Jamovi (Version 2.7.32.0) and R studio (Version 2026.04.0). Participants were stratified into four groups based on their biological sex (male/female) and ASD diagnostic status (ASD/non-ASD). Data discarded at the task level of the CNB were handled using full information maximum likelihood (FIML) estimation during confirmatory factor analysis to yield domain factor scores. Sociodemographics, clinical characteristics, and factor-level cognitive performance, were compared across the four groups using χ2 tests for categorical data and one-way ANOVA for continuous data.

To examine sex differences in neurocognitive performance, a MANCOVA was performed on the three domain-specific CNB/CNB-HK accuracy factor scores (Episodic Memory, Social Cognition and CREF), followed by univariate ANCOVAs for each domain. Both sets of analyses included Group (ASD vs. non-ASD) and Sex as factors with a Group × Sex interaction term. Models were controlled for potential confounders, including age, motor praxis speed, and co-occurring psychopathology (in HATCH: ADHD diagnosis and anxiety symptoms measured by ASC-ASD total score; in PNC: four psychopathology factor scores). In addition, stimulant use during CNB-HK administration was controlled in the HATCH cohort, and race was controlled in the PNC. Post-hoc pairwise comparisons were conducted using Tukey’s HSD test. Statistical significance was set at *p* < 0.05 (two-tailed).

## Results

### Sociodemographic and Clinical Characteristics

In the Hong Kong HATCH cohort, 1,101 Chinese children aged 6–12 years completed the study procedures, including 701 children with ASD (13.4% female) and 400 non-ASD children (44.0% female). Children with ASD were slightly younger than non-ASD children (mean age 8.18 vs. 8.51 years; *p* = 0.028). In the US PNC, females accounted for 21.6% of both the ASD (*n* = 218) and matched non-ASD (*n* = 872) groups, with both groups having a mean age of 12.3 years. Race distribution (*p* = 0.27) was comparable in ASD and non-ASD groups. Parental education levels were also similar across the four groups in both cohorts (*p_HATCH_* = 0.49 and *p_PNC_* = 0.47).

In the HATCH cohort, 47.2% (*n* = 331) of children with ASD had co-occurring ADHD, with 29.2% (*n* = 205) prescribed ADHD medications and 8.4% (*n* = 59) prescribed antipsychotics. Compared with non-ASD children, those with ASD showed significantly more autistic symptoms (SRS-2 total score, *p* < 0.001) and greater co-occurring psychopathologies (CBCL Attention Problem subscale and ASC-ASD total scores, both *p* < 0.001). Children with ASD in this cohort represented a spectrum of clinical severity, with cases distributed across normal to severe/clinical classifications on the SRS-2, CBCL Attention Problems subscale, and ASC-ASD. In the PNC, participants with ASD also exhibited significantly higher scores across all four psychopathology domains (anxious-misery, fear, psychosis, and externalizing behavior; all *p* < 0.001).

FSIQ was assessed only in children with ASD in the HATCH cohort (mean = 97.6, SD = 16.2). In the PNC, the ASD group had a mean estimated IQ by WRAT-4 of 103 (SD = 16.1), which did not differ significantly from the non-ASD group (*p* = 0.592). Therefore, the distribution of IQ in the ASD groups from both cohorts approximated that of the general population. Sociodemographic and clinical characteristics of the study population are summarized in Tables 1 (Hong Kong HATCH cohort) and 2 (US PNC).

### Cognitive Performances of the ASD Participants

Comparisons of cognitive performance between groups were conducted using one-way ANOVA (Table 3). In the HATCH cohort, 104 (14.8%) children with ASD and co-occurring ADHD took stimulants on the day of testing to facilitate completion of the CNB-HK assessment. Children with ASD exhibited slower sensorimotor processing speed on the motor praxis task, with autistic girls showing the slowest performance, followed by autistic boys, non-ASD girls, and non-ASD boys. This aligns with a slower reaction time among ASD individuals [22] and females [26]. They also demonstrated significantly poorer accuracy across all three cognitive domains (CREF, Social Cognition, and Episodic Memory; all *p* < 0.001). In the PNC, participants with ASD similarly showed poorer accuracy in Social Cognition and Episodic Memory (both *p* < 0.001), but performance on CREF was comparable to non-ASD participants (*p* = 0.121).

### Sex Differences in Cognitive Performance in ASD

**Figure 1:**
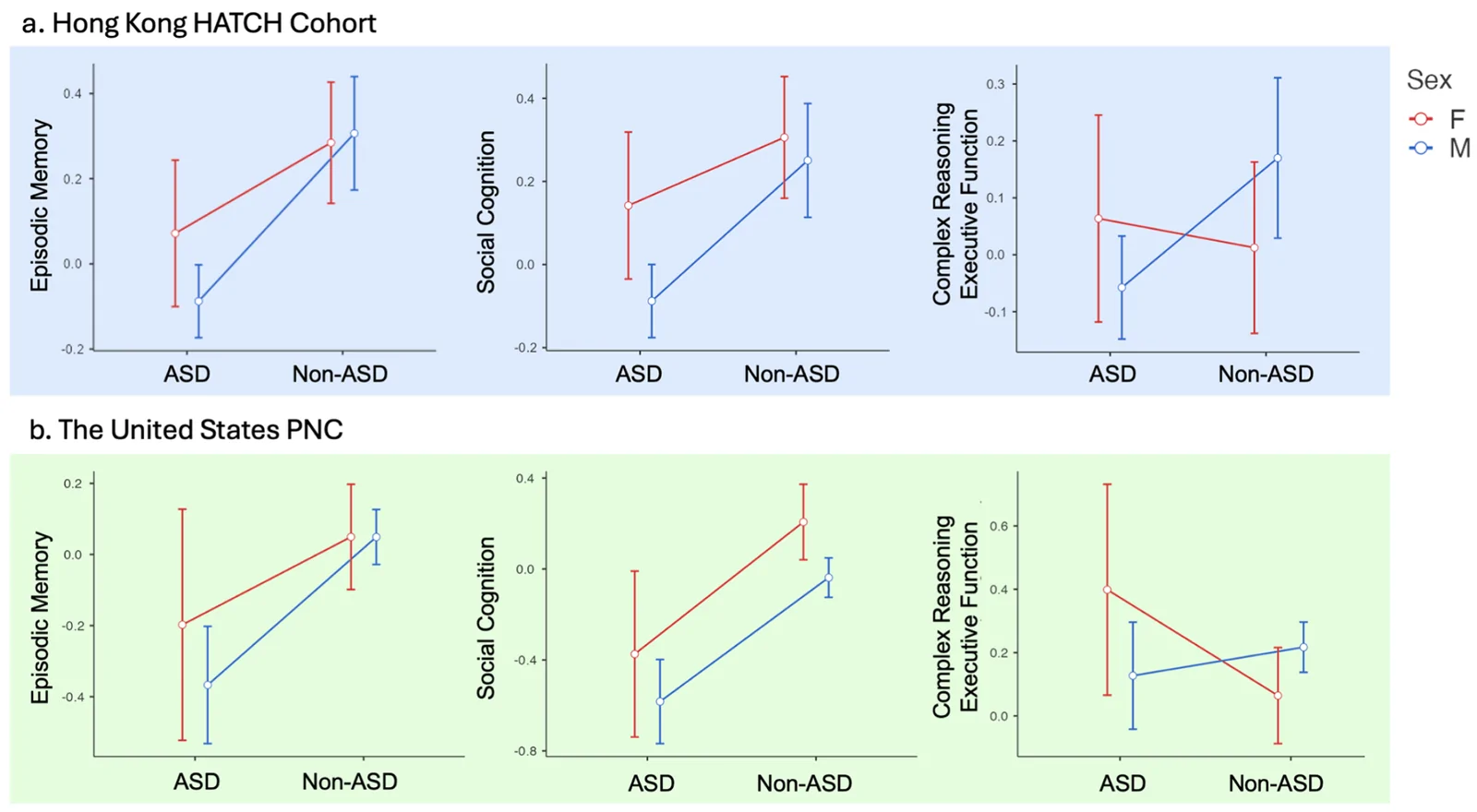
Group × Sex interaction plots for cognitive performance in (a) the HATCH cohort and (b) the US PNC. Estimated marginal means are shown for Episodic Memory, Social Cognition, and Complex Reasoning & Executive Function (CREF) by diagnostic group and sex. Error bars represent 95% confidence intervals.

MANCOVA on the three domain-specific CNB factor scores revealed significant overall effects of Group and Sex in both cohorts (Table 4). The Group × Sex interaction was not significant at the multivariate level in either cohort (Pillai’s Trace 0.005, *p* = 0.144 for HATCH, and Pillai’s Trace 0.007, *p* = 0.092 for PNC). Follow-up univariate ANCOVAs revealed a largely consistent pattern of results across cohorts (Fig. 1). In the domain of Episodic Memory, participants with ASD performed significantly poorer than non-ASD participants in both the HATCH cohort and PNC (both *p* < 0.001, ηp^2^ = 0.066 in HATCH and 0.031 in PNC). A significant main effect of Sex was observed in the HATCH cohort (*p* < 0.001, ηp^2^ = 0.010), with girls outperforming boys, but this effect was not significant in the PNC (*p* = 0.584). In the domain of Social Cognition, males and participants with ASD performed significantly worse in both cohorts (HATCH: both *p* < 0.001, ηp^2^ = 0.047 for Group and 0.017 for Sex; PNC: Group *p* < 0.001, ηp^2^ = 0.041; Sex *p* = 0.005, ηp^2^ = 0.008). However, the Group × Sex interaction was not significant for Episodic Memory (*p_HATCH_* = 0.11, *p_PNC_* = 0.41) or Social Cognition (*p_HATCH_* = 0.14, *p_PNC_* = 0.88) in either cohort, suggesting that any sex differences in these two domains were not specific to the ASD population. In general, effect sizes for group differences were larger in the HATCH cohort compared with the PNC.

In the domain of CREF, a significant main effect of Group was observed in the HATCH cohort (*p* < 0.001, ηp^2^ = 0.033) but not in the PNC (*p* = 0.175, ηp^2^ = 0.002), with no main effect of Sex in either cohort (*p_HATCH_* = 0.68, *p_PNC_* = 0.40). However, a significant Group × Sex interaction was present in both cohorts [HATCH: F(1, 1078) = 5.30, *p* = 0.022, ηp^2^ = 0.005; PNC: F(1, 973) = 4.17, *p* = 0.041, ηp^2^ = 0.004]. In the HATCH cohort, boys with ASD showed the greatest impairment, whereas girls with ASD demonstrated relatively preserved performance (Fig. 1a). This was supported by post-hoc comparisons showing a significant difference between ASD and non-ASD boys (*p_Tukey_* = 0.010, Cohen’s d = 0.28), while girls with ASD performed comparably to non-ASD girls (*p_Tukey_* = 0.969). A similar pattern was observed in the PNC (Fig. 1b), where males with ASD showed numerically lower mean CREF scores and females with ASD appeared relatively preserved or even superior. However, due to the smaller ASD sample size in the PNC, none of the pairwise comparisons reached statistical significance (ASD males vs non-ASD males, *p_Tukey_* = 0.787; ASD females vs non-ASD females, *p_Tukey_* = 0.281).

### Exploratory Analysis Stratifying ASD by Co-occurring ADHD

Given the well-established impact of co-occurring ADHD on executive function in ASD [42], we further conducted an exploratory analysis in the HATCH cohort by stratifying children with ASD into ASD-only and ASD+ADHD subgroups for comparison with non-ASD controls. Similar to the primary analysis, the Group × Sex interaction was not significant at the multivariate level (Pillai’s Trace = 0.01, *p* = 0.32). Yet comparably at the univariate level, a significant Group × Sex interaction was observed for CREF [*F*(2, 1078) = 3.169, *p* = 0.042, ηp^2^ = 0.006], but not for Episodic Memory (*p* = 0.15) or Social Cognition (*p* = 0.22).

**Figure 2:**
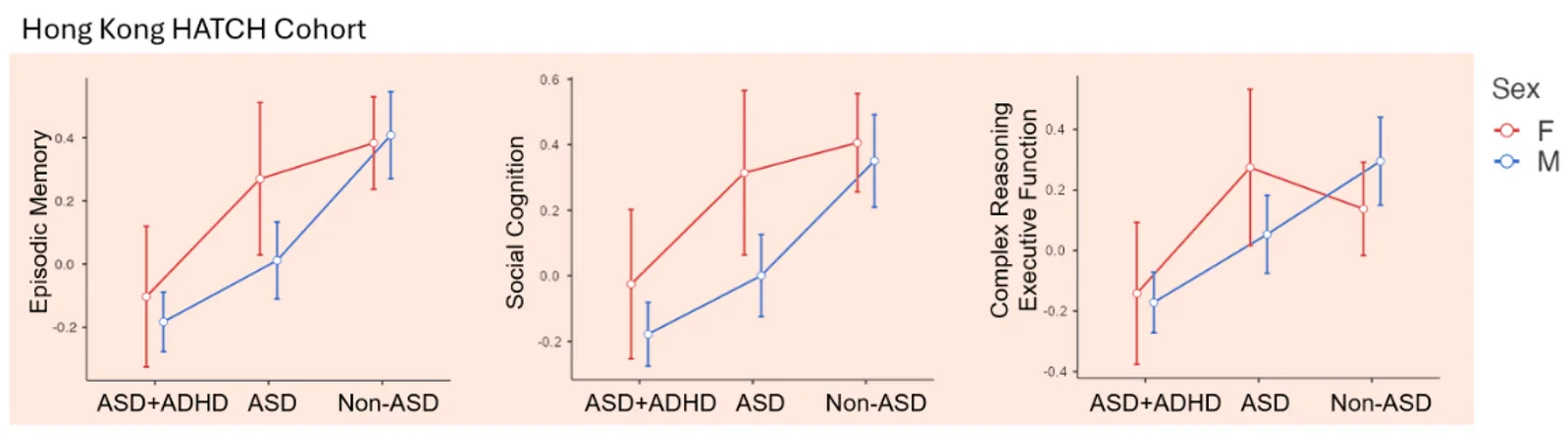
Group × Sex interaction plot for CREF performance across diagnostic subgroups in the HATCH cohort. Estimated marginal means for Complex Reasoning and Executive Function (CREF) are shown for non-ASD controls, ASD-only, and ASD+ADHD groups, stratified by sex. Error bars represent 95% confidence intervals.

The interaction plots (Fig. 2) showed that co-occurring ADHD was generally associated with worse performance across cognitive domains regardless of sex. For Episodic Memory and Social Cognition, performance followed a stepwise decline from non-ASD to ASD-only to ASD+ADHD groups. In contrast, CREF performance in girls with ASD-only was relatively preserved. Post-hoc tests on CREF confirmed a stepwise decline in boys (ASD+ADHD vs. ASD-only, *p_Tukey_* = 0.024, Cohen’s d = 0.28; ASD-only vs. non-ASD, *p_Tukey_* = 0.014, Cohen’s d = 0.30). In girls, ASD-only performed comparably to non-ASD (*p_Tukey_* = 0.922), while ASD+ADHD was associated with numerically lower CREF scores compared with ASD-only girls. This difference did not survive multiple comparison correction (uncorrected *p* = 0.014, *p_Tukey_* = 0.139, Cohen’s d = 0.52), likely due to the modest sample sizes in the female ASD subgroups (ASD-only *n* = 44; ASD+ADHD *n* = 50). Overall, this pattern suggests that co-occurring ADHD may attenuate the female ASD advantage in CREF.

## Discussion

The present study investigated the interplay between sex and ASD on neurocognition. Through comprehensive multi-domain characterisation of cognitive profiles, we found that females with ASD exhibited preserved higher-order cognition, including complex reasoning and executive function, compared to the non-ASD females. The CREF domain in the CNB encompasses tasks that assess visual-spatial skills, reasoning, attention, cognitive flexibility, working memory, and inhibition (see supplementary Table S1). Importantly, this preservation was not observed in Episodic memory or Social Cognition, suggesting that the higher-order cognition specifically differentiates autistic males and females, which may allude to cognitive mechanisms driving sex differences in ASD. Key strengths that our study adds to available literature are a large sample size with rigorous cognitive assessment, the inclusion of a non-ASD comparison group, and statistical adjustment for important demographic and clinical confounders. Notably, the core finding of preserved CREF in autistic females was replicated in an independent US community-based cohort with a broader age range and greater ethnic diversity, suggesting that the results could be generalized across different developmental stages, racial backgrounds, clinical settings, and cultural contexts.

In the domain of Social Cognition, children with ASD consistently showed poorer performance than non-ASD children in both cohorts, with males generally performing worse than females. These findings align with established sex differences in social cognition in the general population [26, 43], and the central role of social cognitive deficits in the everyday social difficulties experienced by autistic individuals [44]. The poorer Episodic Memory in ASD may partly reflect the social component inherent in the face memory task used in this domain, although impaired episodic memory has also been reported in ASD [7]. Overall, social cognitive impairment was evident in both sexes and across the age ranges of both cohorts.

In contrast to the large social cognition effects, the pattern for CREF was more nuanced. In the univariate ANCOVAs following MANCOVA (Table 4), a significant Group effect was observed in the HATCH cohort; this was not replicated in the PNC. This discrepancy may be attributable to differences in control group characteristics. The HATCH non-ASD group was rigorously screened to exclude autism trait and other psychiatric conditions, providing a cleaner disparity with the ASD group. In contrast, the PNC controls were matched on sociodemographic factors but retained dimensional variation in psychopathology. Additionally, the PNC sample was older, and higher-order cognitive abilities may improve with age in ASD [45]. Nonetheless, even in the HATCH cohort where a Group effect was present, the effect size for CREF (ηp^2^ = 0.033) was smaller than those observed for Social Cognition (ηp^2^ = 0.047) and Episodic Memory (ηp^2^ = 0.065). In the PNC, the Group effect on CREF was negligible (ηp^2^ = 0.002). These findings challenge the view that dysfunction of higher-ordered cognition, such as EF, is a ubiquitous feature of ASD [46]. Instead, variability in higher-order cognition may play an important role in explaining the clinical heterogeneity within the autism spectrum, possibly an attribute underlying the sex differences in phenotypic presentation of ASD.

Evidence for sex differences in higher-order cognition in ASD is mixed [17–20, 23, 24] In addition to the aforementioned limitations of existing studies, a recent review specifically highlighted the importance of accounting for co-occurring neurodevelopmental conditions, particularly ADHD, when examining executive function in ASD [47]. Compared with previous work, the present study comprehensively assessed and rigorously controlled for co-occurring psychopathology—including ADHD diagnosis and anxiety symptoms in the HATCH cohort, and dimensional psychopathology factors in the PNC—thereby isolating a sex difference in CREF that is specific to ASD.

Higher-order cognition is postulated to serve as a compensatory mechanism in neurodevelopmental disorders [48]. In ASD, superior executive function may facilitate theory of mind development and camouflaging [49, 50], modulate repetitive behaviours [51], and diminish anxiety reactions associated with sensory hyperresponsiveness [42]. This may explain the qualitative and quantitative characteristics of symptom presentation in the female autism phenotype. However, despite the benefits of optimal higher-order cognition extend beyond core ASD symptoms and are associated with better functional and psychopathological outcomes [12], our findings should be interpreted cautiously. The observed Group × Sex interaction on CREF represents a group-level average with a small effect size, suggesting not all females with ASD have preserved or superior higher-order cognition. This finding aligns with clinical observations that females with ASD could still present with the classical symptoms of motor stereotypies and significantly impaired social-emotional reciprocity. Furthermore, camouflaging is not exclusive to females [52], and even when compensatory strategies are employed, they are not unequivocally beneficial. Camouflaging can incur significant mental health costs, including increased anxiety and emotional exhaustion [53]. Moreover, without any validated screening tools to discern the female autism phenotype, such compensation may contribute to delayed diagnosis in females. Many females with ASD mimic normative social behaviors until the growing complexity of social demands overwhelms their compensatory strategies. They frequently endure significant difficulties before seeking help, often presenting with mood-related concerns, at which point an ASD diagnosis is made retrospectively [54]. This pattern may partly explain recent population-based findings of a decreasing male-to-female ASD incidence ratio from childhood to approaching 1:1 in adulthood [55]. Finally, the observed advantage in CREF was clearest after stringent statistical control for co-occurring psychopathology. In real-world clinical settings, co-occurring psychopathology is highly prevalent in ASD [34], and therefore such advantages cannot be assumed for each individual. As shown in our exploratory analysis, this relative preservation appears diminished when co-occurring ADHD is present.

Although exploratory, the trend that CREF preservation may be lost in females with ASD+ADHD offers a new insight into the female protective effect. These subgroup findings should be interpreted cautiously, however, as they did not survive correction for multiple comparisons. As ADHD is characterized by core deficits in cognitive control [56], it is not surprising that girls with ASD+ADHD in the HATCH cohort showed a trend toward impaired CREF. Given that higher-order cognition supports compensatory mechanisms, females with co-occurring ADHD, and thus more pronounced EF impairment, may be less able to compensate effectively. This is consistent with observations that females with ASD+ADHD exhibit more prominent social communication difficulties than those with ASD alone [57], and being less able to camouflage lead to earlier clinical recognition [58].

This dynamic has implications for genetic studies supporting the female protective effect. Many such studies rely on clinically referred children, for example from the Simons Simplex Collection (SSC)[5, 59] and Autism Genetic Resource Exchange (AGRE) [60]. These cohorts were incepted before the female autism phenotype was widely recognized, and before the DSM-5-TR formally highlighted the need to consider sex differences in presentation in 2022. In addition, passive case-ascertainment methods tend to under-recognize autistic females [1]. As a result, these samples are likely enriched for autistic females who came to clinical attention because of limited capacity to camouflage and/or greater co-occurring conditions such as ADHD.

Given the shared genetic architecture between ASD and ADHD [61], and the involvement of many ASD-associated genes in corticogenesis and cognitive development [62, 63], the apparent higher genetic loading reported in these females may already show up phenotypically as reduced higher-order cognition and greater clinical severity. Supporting this possibility, females with ASD in the SSC showed lower cognitive ability and adaptive skills than males [15]. This suggests that the higher genetic load often reported in autistic females may partly reflect sampling bias toward individuals with poorer cognitive compensation, rather than solely reflecting a higher biological threshold for ASD in females. Future genetic research could address this by active case-finding, include females diagnosed later in life, and systematically account for co-occurring conditions such as ADHD when re-evaluating the female protective effect.

This present study has several limitations. First, the exploratory subgroup analysis of ASD+ADHD was underpowered, particularly for the female subgroups. The observed trend toward impaired CREF in females with ASD+ADHD therefore remains speculative. Second, we did not employ active case-finding strategies. As a result, our samples may not fully capture the entire spectrum of female ASD, particularly those with effective camouflaging who remain undiagnosed or do not present to clinical services. Nonetheless, the HATCH cohort was recruited from a specialist clinic that serves as the main public referral pathway linked to primary care and includes a relatively broad range of presentations, while the community-based, older PNC sample is more likely to include later-diagnosed females and those without co-occurring psychiatric conditions. Third, our assessment of cognitive function relied on laboratory-based performance measures. Although these provide objective data, they may not fully capture everyday executive functioning; parent-reported measures such as the Behavior Rating Inventory of Executive Function (BRIEF) could offer a more ecologically valid and complementary perspective [64]. Fourth, we did not directly measure compensatory strategies such as camouflaging. The role of preserved CREF in compensation is therefore inferred from existing literature, rather than examined in our cohorts. Finally, the cross-sectional design precludes causal inferences regarding the relationship between CREF and sex differences in phenotypic presentation, development or prognosis.

## Conclusion

The present study provides robust evidence that, at the group level, relatively preserved higher-order cognition represents a specific sex difference in ASD. This cognitive pattern may help explain clinical phenotypic differences between males and females, including the less conspicuous female autism phenotype and the tendency for some females to remain unrecognized as ASD during childhood. While preserved higher-order cognition is linked to improved symptoms and functional outcomes, it can also delay diagnosis and incur a later mental health cost when camouflaging becomes unsustainable. These findings, however, reflect group-level differences and should not be regarded as universal. Cognitive ability in females with ASD remain variable and could be modulated by co-occurring conditions. Identification of autistic females must therefore account for this heterogeneity, rather than relying on a single “female autism phenotype”. Future research could move beyond group averages to examine individual differences in cognitive compensation and their links to phenotypic expression and clinical outcomes. Active case-finding, longitudinal follow-ups and consideration of the interplay between higher-order cognition, co-occurring conditions and clinical phenotypes are needed to re-examine the female protective effect. Ultimately, recognizing the unique cognitive profiles in females with ASD can guide development of individualized support that leverages these abilities to enhance adaptation and quality of life.

## Supplementary Material

This is a list of supplementary files associated with this preprint. Click to download.

• SupplementaryMaterialsFemaleASDCognitionv1.docx

## Figures and Tables

**Table 1:** Demographic and Clinical characteristics between the ASD and Non-ASD Participants in the HATCH Cohort

	Hong Kong HATCH Cohort	ASD					Non-ASD					Statistics		
		Male		Female			Male		Female					
	Mean/n	SD/%		Mean/n	SD/%		Mean/n	SD/%	Mean/n	SD/%		F/χ^2^	df	p
n		607	86.59	94	13.41		224	56.00	176	44.00		-		
Age		8.2	1.76	8.2	1.79		8.5	1.74	8.6	1.77		3.07	3, 307	0.028*
Highest parental education attained														
	Primary or less	4	0.66	1	1.06		2	0.89	1	0.57		8.46	9	0.489
	Secondary	208	34.55	37	39.36		68	30.36	52	29.55				
	Tertiary	390	64.78	56	59.57		154	68.75	123	69.89				
Co-occurring ADHD		281	46.29	50	53.19	-					-		
Taking medication														
	ADHD medication	180	29.65	25	26.60		-					-		
	Antipsychotics	52	8.57	7	7.45									
SRS-2 Total Score		68.01	10.17	70.93	11.29		52.14	6.86	53.84	6.83		295.52	3, 313	< 0.001***
	Classification													
	1. Normal	133	22.09	18	19.57		191	86.04	136	78.61		422	9	< 0.001***
	2. Mild	131	21.76	10	10.87		22	9.91	27	15.61				
	3. Moderate	192	31.89	35	38.04		9	4.05	10	5.78				
	4. Severe	146	24.25	29	31.52		0	0.00	0	0.00				
CBCL Attentional Problems Subscale		61.94	10.09	63.92	11.7		52.3	5.24	52.21	4.38		155.25	3, 322	< 0.001***
	Classification													
	1. Normal	428	71.10	57	61.96		217	97.75	170	98.27		144	6	< 0.001***
	2. Boderline	132	21.93	34	36.96		0	0.00	1	0.58				
	3. Clinical	42	6.98	1	1.09		5	2.25	2	1.16				
ASC-ASD Total Score		17.73	10.29	21.77	12.02		9.28	7.23	9.92	7.48		86.41	3, 309	< 0.001***
	Classification													
	1. Normal	369	61.19	44	47.83		205	92.34	155	89.60		130	3	< 0.001***
	2. Elevated	234	38.81	48	52.17		17	7.66	18	10.40				
Full-scale IQ		97.5	16.41	98.46	15.13		-					-		

Note:

* p < 0.05,

*** p < 0.001.

Full-scale IQ was assessed with ASC-ASD: Anxiety Scale for Children - ASD; ADHD: Attention Deficit and Hyperactivity Disorder; CBCL: Child Behavior Checklist; SRS-2: Social Responsiveness Scale, Second Edition

**Table 2.** Demographic and Clinical characteristics between the ASD and Non-ASD Participants in the US PNC

	US PNC	ASD					Non-ASD					Statistics		
		Male		Female			Male		Female					
		Mean/n	SD/%	Mean/n	SD/%		Mean/n	SD/%	Mean/n	SD/%		F/χ^2^	df	p
n		171	78.44	47	21.56		684	78.44	188	21.56		-		
Age		12.18	2.93	12.57	3.37		12.46	3.34	11.88	3.11		1.80	3, 180	0.150
Highest parental education attained														
	Primary or less	0	0	0	0		1	0	0	0		5.59	6	0.470
	Secondary	27	15.90	4	8.70		133	19.50	29	15.60				
	Tertiary	143	84.10	42	91.30		549	80.40	157	84.40				
Race														
	White	129	75.4	38	80.9		524	76.6	151	80.3		7.6	6	0.269
	Black	28	16.4	3	6.4		98	14.3	17	9				
	Others	14	8.2	6	12.8		62	9.1	20	10.6				
Co-occurring Psychopathology														
	Anxious-misery	0.71	1.01	0.36	1.06		−0.13	0.96	−0.09	0.93		32.68	3, 164	< 0.001***
	Psychosis	0.66	0.88	0.41	0.97		−0.07	0.97	−0.16	0.91		34.1	3, 167	< 0.001***
	Externalizing Behaviors	0.76	0.93	0.55	0.96		−0.01	1.01	−0.29	0.92		43.94	3, 168	< 0.001***
	Fear	0.53	0.98	0.2	1.01		−0.2	0.94	0.01	0.92		25.78	3, 164	< 0.001***
Estimated IQ		103	16.31	105	15.61		104.8	16.39	105	14.55		0.637	3,181	0.592

Note:

*** p < 0.001.

IQ was estimated with Wide Range Achievement Test-4 (WRAT-4).

**Table 3:** Comparison of Neurocognitive Performance in the Hong Kong HATCH cohort and US PNC

Task/Factor	Hong Kong HATCH cohort					US PNC					Statistics (HK)					Statistics (US)			
	ASD		Non-ASD			ASD		Non-ASD											
Male	Female	Male	Female		Male	Female	Male	Female		F	df	p	ηp^2^		F	df	p	ηp^2^	
Motor Praxis	−0.24(1.23)	−0.61 (1.55)	0.21 (0.83)	−0.02 (0.98)		0.74 (2.73)	0.10 (2.49)	0.31 (1.74)	0.38 (1.60)		16.05	3, 311	< 0.001***	0.134		1.3	3, 145	0.277	0.026
CREF	−0.21 (0.92)	−0.08 (0.87)	0.28 (0.75)	0.07 (0.67)		−0.05 (1.28)	0.15 (1.38)	0.20 (1.01)	0.11 (0.94)		13.62	3, 311	< 0.001***	0.113		1.97	3, 161	0.121	0.035
Social Cognition	−0.18 (0.89)	−0.01 (0.87)	0.24 (0.67)	0.26 (0.65)		−0.63 (1.33)	−0.54 (1.41)	−0.07 (1.07)	0.23 (1.09)		25.52	3, 321	< 0.001***	0.193		15.79	3, 161	< 0.001***	0.228
Episodic Memory	−0.19 (0.88)	−0.09 (0.79)	0.30 (0.65)	0.23 (0.63)		−0.49 (1.13)	−0.29 (1.38)	0.02 (0.96)	0.09 (0.95)		31.12	3, 324	< 0.001***	0.224		11.44	3, 161	< 0.001***	0.176

Note.

*** p < 0.001.

Values are presented as Mean (SD).

CREF = Complex Reasoning and Executive Function

**Table 4.** MANCOVA Results for Neurocognitive Domains in the Hong Kong HATCH cohort and the US PNC

Multivariate Tests		Hong Kong HATCH			US PNC		
	Effect	Pillai’s Trace	F (df)	p	Pillai’s Trace	F (df)	p
	Group	0.066	25.20 (3, 1076)	< 0.001***	0.07	24.46 (3, 971)	< 0.001***
	Sex	0.031	11.58 (3, 1076)	< 0.001***	0.016	5.39 (3, 971)	0.001**
	Group × Sex	0.005	1.81 (3, 1076)	0.144	0.007	2.15 (3, 971)	0.092
Univariate tests							
Cognitive Domain	Effect	F (df)	p	ηp^2^	F (df)	p	ηp^2^
Episodic Memory	Group	75.62 (1, 1078)	< 0.001***	0.066	31.31 (1, 973)	< 0.001***	0.031
	Sex	11.05 (1, 1078)	< 0.001***	0.01	0.30 (1, 973)	0.584	0.001
	Group × Sex	2.51 (1, 1078)	0.114	0.002	0.70 (1, 973)	0.405	0
Social Cognition	Group	52.83 (1, 1078)	< 0.001***	0.047	41.3 (1, 973)	< 0.001***	0.019
	Sex	18.76 (1, 1078)	< 0.001***	0.017	7.81 (1, 973)	0.005**	0.008
	Group × Sex	2.19 (1, 1078)	0.139	0.002	0.02 (1, 973)	0.876	0
CREF	Group	37.02 (1, 1078)	< 0.001***	0.033	1.84 (1, 973)	0.175	0.002
	Sex	0.17 (1, 1078)	0.677	0	0.70 (1, 973)	0.401	0.001
	Group × Sex	5.30 (1, 1078)	0.022*	0.005	4.17 (1, 973)	0.041*	0.004

Note:

* p < 0.05,

** p < 0.01,

*** p < 0.001.

Hong Kong HATCH models controlled for age, anxiety symptoms (ASC-ASD total score), ADHD diagnosis, stimulants use during CNB, and motor praxis speed. US PNC models controlled for age, race, four psychopathology factors (Anxious-Misery, Fear, Psychosis and Externalizing Behaviour), and motor praxis speed.

## Data Availability

The datasets used and/or analysed during the current study are available from the corresponding author on reasonable request

## Abbreviations

ADHD: Attention–Deficit/Hyperactivity Disorder
ADI: R–Autism Diagnostic Interview–Revised
ADOS: Autism Diagnostic Observation Schedule
ANCOVA: Analysis of Covariance
AQ: 10–HK–Hong Kong version of the 10–item Autism Spectrum Quotient
ASC: ASD–Anxiety Scale for Children–Autism Spectrum Disorder
ASD: Autism Spectrum Disorder
BRIEF: Behavior Rating Inventory of Executive Function
CBCL: Child Behavior Checklist
CNB: Penn Computerized Neurocognitive Battery
CNB: HK–Hong Kong version of the Penn Computerized Neurocognitive Battery
CREF: Complex Reasoning and Executive Function
DISC: 5–Diagnostic Interview Schedule for Children, Version 5
DSM: 5–Diagnostic and Statistical Manual of Mental Disorders, Fifth Edition
EF: Executive Function
FIML: Full Information Maximum Likelihood
FSIQ: Full–Scale Intelligence Quotient
GOASSESS: Grand Opportunity Assessment
HATCH: Hub of Advanced Technology for Child Health
MANCOVA: Multivariate Analysis of Covariance
PNC: Philadelphia Neurodevelopmental Cohort
RRB: Restricted and Repetitive Behaviors
SRS: 2–Social Responsiveness Scale, Second Edition
SSC: Simons Simplex Collection
WRAT: 4–Wide Range Achievement Test, Fourth Edition
WISC: IV (HK)–Hong Kong Wechsler Intelligence Scale for Children, Fourth Edition
